# Acute spontaneous subdural hematoma caused by skull metastasis of hepatocellular carcinoma: case report

**DOI:** 10.1186/s12893-015-0045-x

**Published:** 2015-05-10

**Authors:** Cien-Leong Chye, Kuo-Hsuan Lin, Chang-Hsien Ou, Cheuk-Kwan Sun, I-Wei Chang, Cheng-Loong Liang

**Affiliations:** Department of Neurosurgery, E-Da Hospital, School of Medicine, I-Shou University, 1 E-Da Rd., Yan-Chau Dist, 824 Kaohsiung City, Taiwan; Department of Emergency Medicine, E-Da Hospital, School of Medicine, I-Shou University, Kaohsiung, Taiwan; Department of Diagnostic Radiology, E-Da Hospital, Kaohsiung, Taiwan; Department of Pathology, E-Da Hospital, Kaohsiung, Taiwan

**Keywords:** Hepatocellular carcinoma, Metastasis, Subdural hematoma

## Abstract

**Background:**

Skull and intracranial metastases from hepatocellular carcinoma (HCC) have seldom been reported. A skull metastasis of HCC with a tumor bleeding resulting in spontaneous subdural hematoma (SDH) is extremely unusual. We report the first case of acute spontaneous SDH in a 69-year-old woman who presented with acute onset of headache, because of tumor bleeding caused by skull metastasis of HCC.

**Case presentation:**

A 69-year-old woman was referred to our hospital because of progressive headache, nausea, and vomiting for 3 days. Brain computed tomography (CT) performed in the emergency department (ED) revealed a left temporal SDH with a slight mass effect and a small left temporal bone erosion. Tri-phasic abdominal CT demonstrated a large right lobe liver tumor compatible with HCC. She experienced progressive deterioration of consciousness in the intensive care unit. Follow-up CT showed an enlargement of the SDH. An emergency craniotomy for hematoma evacuation and removal of skull tumor was performed. She regained consciousness and had no neurological deficits during the postoperative course. Pathological examination of the skull specimen indicated metastasis of a HCC.

**Conclusion:**

Patients with acute SDH without a history of head injury are rarely encountered in the ED. Metastatic carcinoma with bleeding should be included as a differential diagnosis for acute spontaneous SDH. Before an operation for SDH, the possibility of metastatic lesion of the skull should be considered in the surgical planning and the origin of malignancy should be sought.

## Background

Acute subdural hemorrhage (SDH) commonly results from closed head injury. The linear translation of acceleration along the skull can cause injury to the veins, arteries, or brain parenchyma, resulting in SDHs, epidural hematomas, or contusion hematomas [1]. Skull and intracranial metastases from hepatocellular carcinoma (HCC) have seldom been reported [2,3]. The typical metastatic sites of HCC are regional lymph nodes and the lungs [4]. A skull metastasis of HCC with a tumor bleeding resulting in spontaneous SDH is extremely unusual. We report a case of acute spontaneous SDH in a 69-year-old woman who presented with acute onset of headache, because of tumor bleeding caused by skull metastasis of HCC.

## Case presentation

**Fig. 1:**
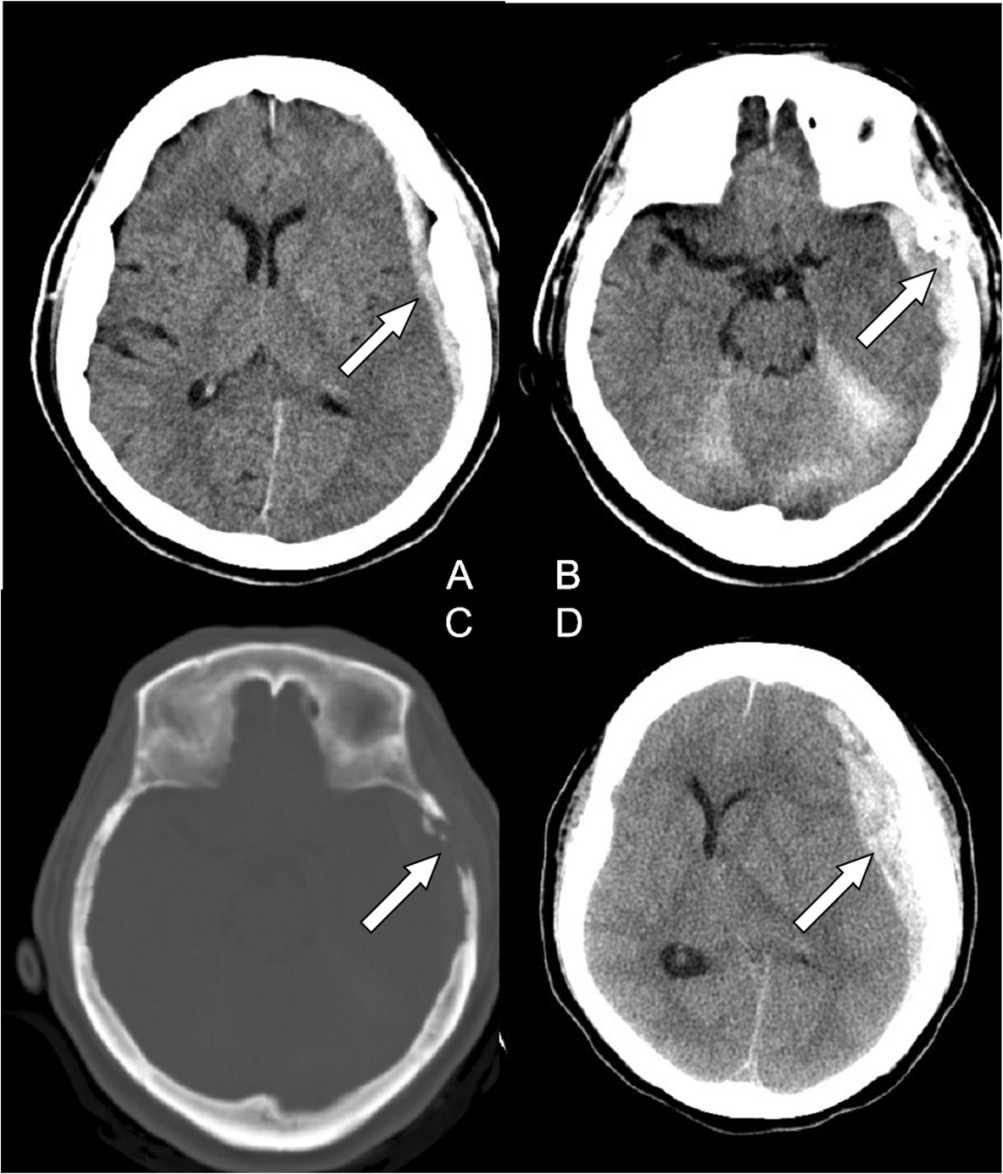
**(a)** Computed tomography (CT) of the head showing left temporal subdural hematoma (SDH) (arrow) with slight mass effect. **(b)** small left temporal bone erosive lesion (arrow). **(c)** A bony defect on the left temporal bone (bone window of CT) (arrow). **(d)** Enlargement of left SDH with significant mass effect (arrow)

**Fig. 2:**
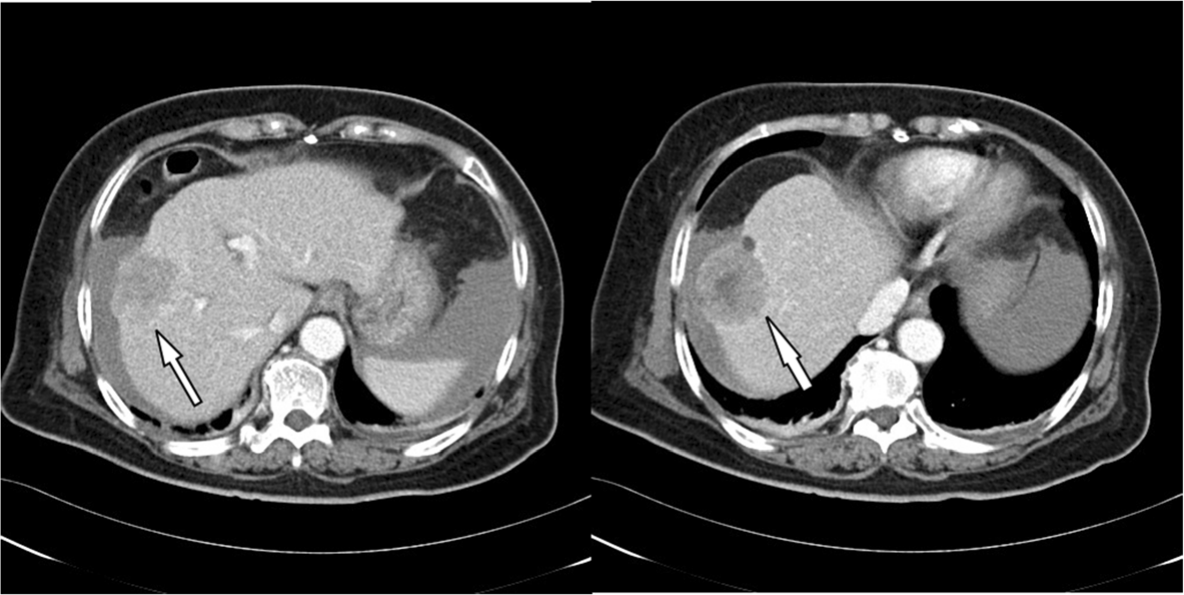
Tri-phasic abdominal CT revealing a large tumor (4.8 cm in diameter) over segment 8 of right lobe compatible with hepatocellular carcinoma (arrow)

A 69-year-old woman was referred to our hospital because of progressive headache, nausea, and vomiting for 3 days. In addition, she complained of dizziness and a decrease in appetite. She had neither systemic disease nor history of recent trauma or injury to the head. Her Glasgow Coma Scale (GCS) was 13 (E3V4M6) at the emergency department (ED) without notable focal neurological signs. Routine laboratory examination demonstrated normal liver function with no evidence of coagulopathy and thrombocytopenia. Chest radiography showed no remarkable findings. However, emergency computed tomography (CT) of her head revealed a left temporal SDH with slight mass effect and small left temporal bone erosion (Fig. 1a-c). Metastasis tumor was highly suspected. The additional diagnostic work up with tri-phasic abdominal CT demonstrated a large right lobe liver tumor compatible with HCC (Fig. 2). Examination of the serum tumor markers revealed a marked elevation of α-fetoprotein (3815.14 ng/mL). She was admitted to ICU for observation and further evaluation. The blood test to determine the risk of HCC which is related to virus infection revealed positive for hepatitis C virus antigen.

**Fig. 3:**
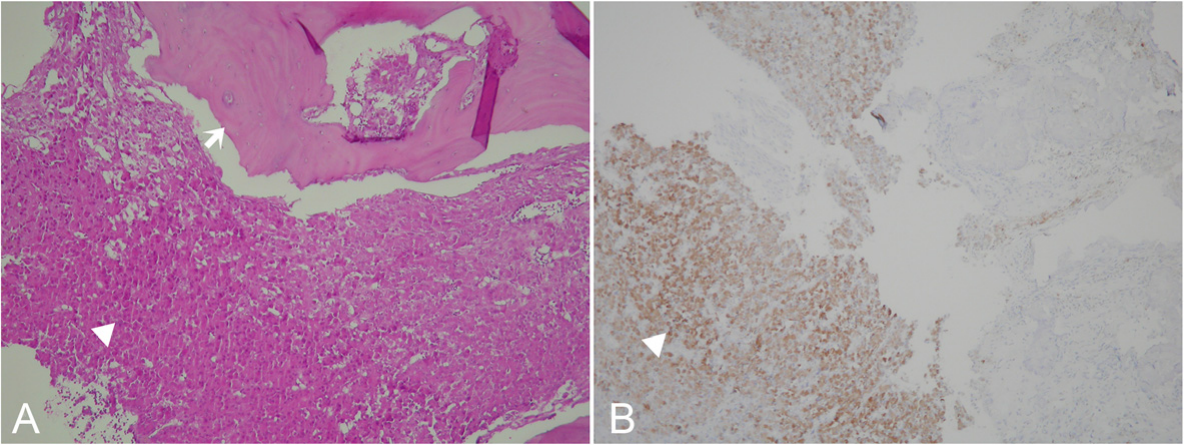
Histologic features of tumor. **a**: Under 200x magnification, the microscopic view with Hematoxylin and eosin (H&E) staining, sections show high cellularity tumors with trabecular-pattern arrangement (arrow head), and tumor cells with bone involvement (arrow) **b**: Under 200x magnification, the microscopic view with immunohistochemical staining, tumor cells were immunoreactive to Hep par-1 (arrow head), an antibody which is highly selective for hepatocellular carcinoma

During her second day in the ICU, her consciousness deteriorated significantly. Her GCS dropped from 13 to 8 and her pupils were anisocoric. Emergency brain CT showed an enlargement of the left temporal SDH with significant mass effect (Fig. 1d) for which an emergency craniotomy for SDH evacuation and removal of metastatic skull tumor were performed. During the operation, a left temporal bone tumor was found to involve the dura causing a dural defect. These findings are compatible with tumor bleeding with hematoma ruptured into subdural space. HCC with skull metastasis was pathologically confirmed using the skull specimen (Fig. 3). After operation, the patient regained consciousness and had no neurological deficits. The patient was transferred to the department of oncology for transcatheter arterial chemoembolization for HCC and brain radiotherapy.

### Discussions

Skull metastasis from HCC occurred infrequently, in about 0.4–1.6% of patients with HCC [2]. HCC commonly metastasizes to regional lymph nodes and the lungs, but rarely to the skeletal system. The most common sites of bone metastasis are the vertebrae, followed by the pelvis and ribs [3]. The incidence of bone metastases from HCC accounts for approximately 1.6–16% [4]. The current literature indicates that skull and intracranial metastases from HCC are uncommon [3,4]. Moreover, cases of intracranial hemorrhage resulting from metastasis of HCC are even rarer [2,5].

To our best knowledge, there is no report of acute SDH from a metastatic skull lesion of HCC. Hsieh et al. reviewed 68 patients with HCC and skull metastases [3]. They reported that the most common presentation is a subcutaneous mass with an occasional painful sensation, accounting for 59% of the patients, while cranial nerve deficits, such as visual disturbance, deafness, and facial numbness can be principal manifestations when the skull base is involved. In our patient, the initial symptoms were sudden headache, dizziness, and nausea without neurological deficits. The progressive mass effect from acute SDH caused a deterioration of consciousness. The most common hemorrhagic metastatic brain tumors include melanomas, choriocarcinomas, renal cell carcinomas, and bronchogenic carcinomas [6]. Although metastatic brain tumors may present with spontaneous intracerebral hemorrhage, SDH is very uncommon. Acute spontaneous SDH may occur in a patient receiving anticoagulation therapy, regardless of a history of trauma. Receiving anticoagulation therapy increases the risk of acute SDH 7-fold in males and 26-fold in females [7]. Overall, the majority of the identifiable causes of acute spontaneous SDH are arterial (61.5%), then idiopathic (10.8%), coagulopathic (10.1%), spontaneous intracranial hypotension (5.4%) and neoplastic (5.4%) [8].

Anatomically, the dura mater is a thick, dense and fibrous membrane. It can be devided to outer endosteal layer and inner meningeal layer. Microscopically, the dura was found to consist predominantly of collagen fibers, although the thickness of the dura varied between sites. Dura was significantly thinner in relation to the ethmoid sinus, tegmen and sigmoid sinus, indicating its greater susceptibility to possible injury at these sites during surgery and head trauma [9,10]. The thinnest dural mater mostly located in skull base and sinus areas. Skull metastatic tumor could invade through the thin dura directly to subdural space, and consequently tumor bleeding might cause subdural hematoma.

Our patient presented with a sudden onset of headache that resulted from spontaneous SDH presumably due to the bleeding of the metastatic tumor that passed through the dural perforation into the subdural space. We emphasize that if an acute SDH is found in a patient without a history of trauma, then skull metastasis with tumor bleeding should be considered. The possibility of malignancy with skull metastases can guide an appropriate surgical strategy. Surgical planning of metastatic tumor bleeding is different from traumatic SDH. Total removal of metastatic tumor and evacuation of SDH are equally the top priority of surgical planning.

## Conclusion

Acute spontaneous SDH in patients without a history of head injury is rarely encountered. Metastatic carcinoma with a tumor bleeding should be included in the differential diagnosis for patients presenting with spontaneous SDH.

## Consent

Written informed consent was obtained from the patient’s son for publication of this case report and any accompanying imagines. A copy of the written consent is available for review by the Editor of this journal.

## Abbreviations

HCC: Hepatocellular carcinoma
SDH: Subdural hematoma
ED: Emergency department
CT: Computed tomography
ICU: Intensive care unit
